# Strenuous 12-h run elevates circulating biomarkers of oxidative stress, inflammation and intestinal permeability in middle-aged amateur runners: A preliminary study

**DOI:** 10.1371/journal.pone.0249183

**Published:** 2021-04-01

**Authors:** Ewa Sadowska-Krępa, Michał Rozpara, Adam Rzetecki, Sebastian Bańkowski, Aleksandra Żebrowska, Wanda Pilch

**Affiliations:** 1 Institute of Sport Sciences, Jerzy Kukuczka Academy of Physical Education, Katowice, Poland; 2 Department of Chemistry and Biochemistry, Institute for Basic Science, University of Physical Education, Krakow, Poland; Universidade Federal de Minas Gerais, BRAZIL

## Abstract

Given the solid evidence that prolonged strenuous exercise is a cause of metabolic stress, this study sought to determine whether a 12-h run would affect total oxidant status (TOS), total oxidant capacity (TOC), total antioxidant status (TAS), high-sensitivity C-reactive protein (hs-CRP) and the biomarkers of intestinal permeability (protein fatty acid-binding proteins (I-FABP) and zonulin) in middle-aged male subjects. Ten amateur long-distance runners (aged 52.0 ± 6.2 years, body height 176.9 ± 4.9 cm, body mass 73.9 ± 6.0 kg) were enrolled in the study. The venous blood samples were collected 1 hour before and right after the run and were analyzed for the levels of TAS, TOS/TOC, hs-CRP, I-FABP and zonulin. The post-run concentrations of TOS/TOC were significantly elevated (p < 0.001), but TAS changes were not significant. Pearson’s correlation coefficients calculated for the post run values of TAS and TOS/TOC were statistically significant and negative (r = -0.750, p < 0.05). Significant increases in the concentrations of hs-CRP (p < 0.001), I-FABP (p < 0.05) and zonulin (p < 0.01) were noted. The results indicate that a strenuous 12-h run disturbs the prooxidant-antioxidant balance in middle-aged men, as well as promoting inflammation and impairing intestinal permeability.

## Introduction

Long-distance running is a very popular form of physical activity, but there is much evidence that prolonged strenuous exercise leads to metabolic stress [1] and promotes the production of reactive oxygen species (ROS), mainly by increasing electron leakage from the respiratory chain, auto-oxidation of hemoglobin and catecholamines, and the activity of xanthine oxidase during reperfusion and of NADPH oxidase in response to inflammation caused by microinjuries within the skeletal muscles [2]. Excessive production of ROS causes oxidative damage to proteins, lipids, and nucleic acids [3]. In assessing the level of oxidative stress in biological fluids, total oxidant status and total oxidant capacity (TOS/TOC) showing the total lipid peroxide concentration directly related to the level of oxygen radicals are used more and more often [4, 5].

The exercise-induced production of ROS plays a major role in progression of inflammation. ROS produced at the inflamed site cause the dysfunction of the vascular endothelium and contribute to the transmigration of inflammatory cells through the endothelial barrier, which leads to tissue damage [6]. The presence of inflammation can be determined using blood circulating C-reactive protein (CRP), whose concentration increases very fast, even within several hours from the onset of inflammation [7].

Running at 80% VO_2max_ [8] and prolonged strenuous exercise (≥2 hours at 60% of VO_2max_) are known to cause digestive tract disfunction [9] that induce a variety of gastrointestinal responses in the athletes. Loose stool, nausea, vomiting, diarrhea, urinary incontinence, and rectal hemorrhage have been observed after the run in almost half of long-distance runners [10, 11]. Other frequent problems include mucosal erosion and ischemic intestinal inflammation [12, 13]. One cause of the post-exercise gastrointestinal problems is ischemia-reperfusion injury (IRI) resulting from a temporary disruption of splanchnic blood flow. As strenuous exercise ends, the previously hypoxic tissues receive a large influx of oxygen, which triggers ROS production, inflammation, and causes damage to gastrointestinal mucosa [12, 14, 15].

Intestinal permeability can be assessed using a range of biomarkers, including intestinal fatty acid-binding protein (I-FABP) [16] and zonulin [17].

I-FABP is a protein that binds fatty acids into long chains and transports them to the sites of intracellular utilization. It is mainly produced by mature enterocytes in the small intestine. Because damaged intestinal mucosa releases I-FABP to the bloodstream, its elevated concentration in serum is considered be indicative of increased intestinal permeability [16, 18].

Zonulin, a haptoglobin-related protein, is also frequently used in clinical practice to assess intestinal permeability [17]. In most cases, its concentration is determined using stool or blood samples. Zonulin modulates the tight junctions (TJ) that under physiological conditions seal the paracellular spaces between enterocytes and regulate the transport of fluids, macromolecules and leukocytes between the intestinal lumen and the bloodstream, as well as intestinal permeability. An elevated serum concentration of zonulin indicates decreased intestinal integrity [19–21].

Metabolic changes induced by prolonged strenuous exercise such as marathon or ultra-marathon running are well documented in the literature [1, 22, 23], but there is a research gap regarding the metabolic effects of a 12-h run, despite the growing interest in this type of physical activity [24]. As our study was designed to determine the effect of a long-distance run on the levels of selected biomarkers in the middle-aged male runners, we tested a hypothesis that metabolic stress induced by a 12-h run would disturb their prooxidant-antioxidant balance, as well as causing inflammation and increasing the levels of intestinal permeability markers in serum.

## Materials and methods

### Participants

Seventy-four male athletes intending to participate in a 12-h run organized by the Municipal Sports and Recreation Canter and the recreation and sports club TKKF “Jastrząb” in Ruda Śląska (Poland) were screened for the study. Fifty of them met the inclusion criteria, i.e., were male, aged ≥ 35 years, had a training history ≥ 3 years, and did not intend to use non-steroidal analgesics before and during the race. Of the 50 selected athletes, only 10 agreed to participate and were enrolled in the study (mean age 52.0 ± 6.2 years, body height 176.9 ± 4.9 cm, body mass 73.9 ± 6.0 kg, running a distance of 57.4 ± 22.9 km weekly, with a training history of 7.3 ± 2.2 years).

All participants filled out the medical and training history questionnaires, signed the consent forms, and were briefed on the study protocol that conformed to the ethical guidelines of the Declaration of Helsinki and was approved by the Local Bioethics Committee at the Jerzy Kukuczka Academy of Physical Education in Katowice (certificate of approval No. KB/13/17).

### Study circumstances

The run, which took place between 7 a.m. and 7 p.m., on April 28, 2018, in the town of Ruda Śląska, required the contestants to run a circular route of 1.6 km as many times as they could. The mean air temperature during the day was 24 ± 4°C and relative humidity 46 ± 2%. The runners ingested carbohydrate-rich food (sandwiches, cookies, fruits, and carbohydrate energy bars) every 90–120 min and fluids (water and sport beverages) every 20–25 min. According to the event rules, they could take short rest breaks at their discretion.

### Biochemical analyses

Venous blood samples were collected 1 hour before the run and immediately after it ended. One part of a sample was placed in the hematocrit capillary tube and the other part in test tubes for separation of plasma (BD Vacutainer® PPT™ Plasma Preparation Tube, UK) and serum (BD Vacutainer™ Serum Tube, UK). Hematocrit (HCT) was measured by micro-hematocrit method (Hettich 210, DJB Labcare, UK). Plasma was separated by centrifugation for 10 min at 1000 × g at 4^◦^C (SIGMA 2-16KL, Sigma Laborzentrifugen GmbH, Germany). The serum tubes were allowed to stand for 30 min for blood to clot before serum was separated. Plasma and serum were then stored frozen for analysis at -80^◦^C for a period shorter than one month without unfreezing and freezing.

The plasma concentration of lactate (LA) and total antioxidant status (TAS) were measured using a colorimetric method (a Randox Laboratories Ltd. diagnostic kit UK). Plasma total oxidant status/total oxidant capacity (TOS/TOC) were assessed by a photometric method (a PerOx test kit from Immundiagnostik AG, Germany). The intra- and inter-assay coefficients of variation (CV) were 0.86% and 3.62% (LA), 1.33–5.06% and 3.04–4.07% (TAS), and 2.94% and 6.63–6.85% (TOS/TOC). The reference ranges for TOS/TOC–< 200 μmol/l, 200–350 μmol/l, and > 350 μmol/l–correspond to low, moderate, and high oxidative stress, respectively. The serum concentration of high-sensitivity C-reactive protein (hs-CRP) was measured with a commercially available kit (Dade Behring, Marburg, Germany; the intra-assay CV: 4.4%; the inter-assay CV: 5.7%). The serum concentrations of fatty acid-binding proteins (I-FABP) and zonulin were determined by means of the human I-FABP ELISA kit (Hycult®Biotech, Netherlands) and the IDK®Zonulin ELISA kit (Immundiagnostik AG, Germany The intra- and inter-assay CVs were 3.2–6.6% and 0.2–1.9% (I-FABP), 3.5–6.0% and 7.7–8.3% (zonulin).

The oxidative stress index (OSI) was defined as a ratio between TOS/TOC and TAS; where both indices were expressed in μmol/l after converting TAS from mmol/l to μmol/l [25].

All biochemical tests were performed as per PN-ENISO 9001:2015 (certificate no. PW-19912-18B) and the test manufacturers’ instructions by a certified laboratory.

The values of the biomarkers were adjusted for exercise-induced dehydration. A two-step procedure was used to this end. First, the percentage change in plasma volume (%ΔPV) was calculated with the following formula: %ΔPV = [100 / (100 –HCT1)] × [100 (HCT1 –HCT2) / HCT2], where HCT1 –hematocrit before the run, HCT2 –hematocrit after the run [26]. Then, the indicators’ values were corrected using a formula proposed by Kraemer and Brown [27]–CV = (%ΔPV × 0.01 × V2) + V2, where CV–a corrected value, V2 –a post-run value.

### Statistical analysis

The data analyzed below represent mean values (M), standard deviations (SD), mean differences (MD), standard deviation differences (SD_D_), and confidence interval (CI). The data normality distribution was established using the Shapiro-Wilk test. The significance of differences within-subjects was assessed with a paired-samples t-test. The effect size for the paired-samples t-test was estimated by calculating Cohen’s d index (d_c_) [28]. The correlations between the selected variables were evaluated with Pearson’s correlation coefficient, and power analysis for paired sample t-test was performed (1-β). The level of significance in all tests was set to α = 0.05. The statistical analysis was performed in IBM SPSS Statistics 26.0 (IBM Corporation, Armonk, NY, USA) and G*Power 3.1 [29].

## Results

Measurements made immediately after the run during which the study participants ran an average distance of 94.73±12.97 km (min–max: 72.80–113.60 km) with a speed 7.89±1.07 km/h (min–max: 6.10–9.50 km/h). At a ΔPV of -2.37 ± 9.25%, statistically significant increases were observed in the levels of LA (p < 0.001), TOS/TOC (p < 0.001), and OSI (p < 0.001) (Table 1).

**Table 1:** The pre- and post-run values of lactate and the biomarkers of prooxidant-antioxidant balance (n = 10).

Variable	M ± SD		MD ± SD_D_	±95% CI	d_c_	1-β
	pre-run	post-run				
LA, mmol/l	1.90 ± 0.55	4.84 ± 1.05***	2.95 ± 1.17	2.11, 3.78	3.51	1.00
TOS/TOC, μmol/l	265.11 ± 107.90	573.33 ± 129.76***	308.22 ± 158.00	195.05, 421.24	2.58	1.00
TAS, mmol/l	1.50 ± 0.08	1.52 ± 0.12	0.02 ± 0.07	-0.03, 0.07	0.14	0.15
OSI	0.18 ± 0.07	0.37 ±0.10***	0.19 ± 0.11	0.12, 0.27	2.18	1.00

Note: M–a mean; SD–standard deviations; MD–a mean difference; SD_D_−a standard deviation difference; ±95% CI–confidence interval for the difference between two means; d_c_−Cohen’s d with correction, Dunlap et al. [28]; 1-β–observed (post-hoc) power.

Differences significant at

* p ≤ 0.05

** p ≤ 0.01

*** p ≤ 0.001.

**Fig 1:**
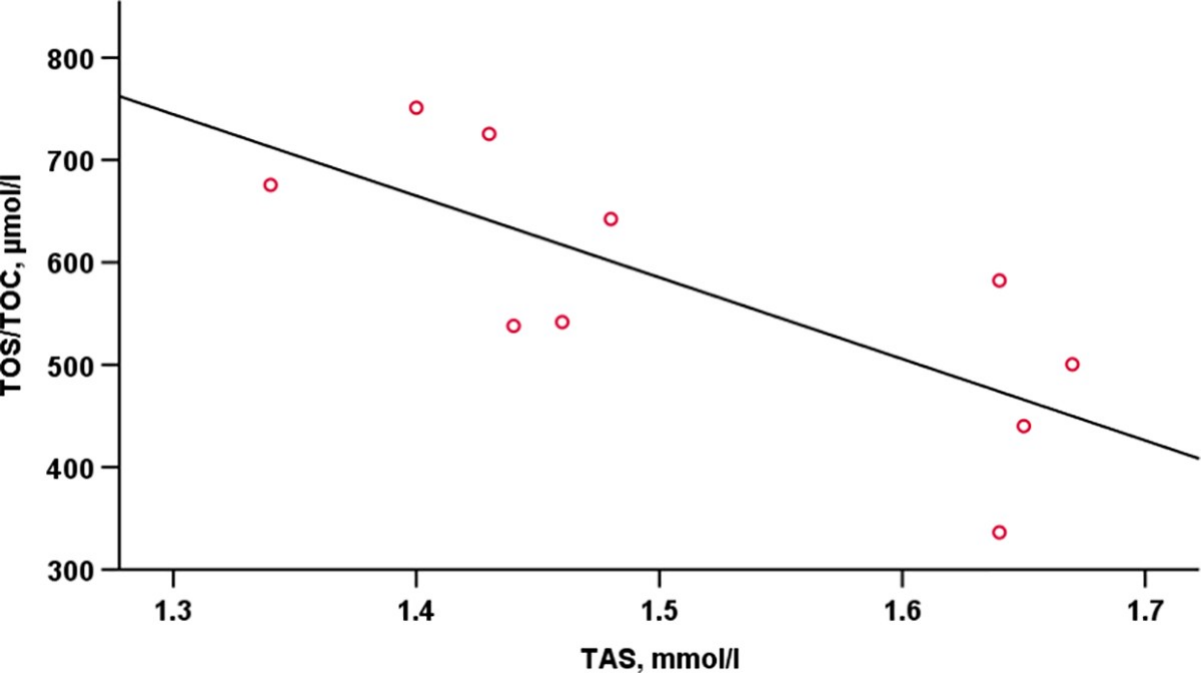
Correlations between the post-run TAS and TOS/TOC.

Pearson’s coefficients of correlations between the post-run TAS and TOS/TOC were statistically significant and negative (r = - 0.750, p < 0.05) (Fig 1).

Increases in the concentrations of hs-CRP (p < 0.001), I-FABP (p < 0.05), and zonulin (p < 0.01), measured post-run were also statistically significant (Table 2).

**Table 2:** The pre- and post-run values of C-reactive protein and intestinal permeability biomarkers (n = 10).

Variable	M ± SD		MD ± SD_D_	±95% CI	d_c_	1-β
	pre-run	post-run				
hs-CRP, mg/l	1.64 ± 0.88	7.90 ± 4.20***	6.26 ± 3.69	3.62, 8.91	1.43	1.00
I-FABP, pg/ml	176.16 ± 87.44	396.70 ± 240.35*	220.54 ± 221.24	62.27, 378.80	1.10	0.80
zonulin, ng/ml	58.45 ± 10.55	65.55 ± 12.53**	7.10 ± 6.41	2.51, 11.69	0.59	0.87

Note: M–mean; SD–a standard deviation; MD–a mean difference; SD_D_−a standard deviation difference; ±95% CI–confidence interval for the difference between two means; dc–Cohen’s d with correction, Dunlap et al. [28]; 1-β–observed (post-hoc) power.

Differences significant at

* p ≤ 0.05

** p ≤ 0.01

*** p ≤ 0.001.

## Discussion

Knowing that strenuous exercise leads to metabolic stress we decided to investigate whether a 12-h run will affect biomarkers of oxidative stress, inflammation and intestinal permeability in middle-aged amateur runners.

There is solid evidence that aerobic exercise disturbs the prooxidant-antioxidant balance in the skeletal muscles and blood [2]. In our study, the TOS/TOC measured pre-run pointed to moderate oxidative stress, as could be expected in well-trained individuals, but their post-run values indicated high oxidative stress. Another evidence of the prooxidant-antioxidant balance having been disturbed by strenuous 12-hour run was a negative correlation between TOS/TOC and TAC and a significant increase in OSI. These results are consistent with other studies reporting an imbalance between oxidant and antioxidant protection in marathon runners post-run [23, 30].

Oxidative stress can cause inflammation by activating various transcription factors, which results in the expression of inflammatory pathway genes [31]. Systemic low-grade inflammation caused by bacterial infections, injuries, tissue necrosis [7, 32], gastrointestinal diseases (e.g. Crohn’s disease), or acute pancreatitis [33] can be reliably detected by CRP. In healthy adults, the concentration of CRP ranges from 0.5 to 5.0 mg/l, but inflammation may increase it from several hundredfold to thousandfold [32, 34]. Unlike single physical effort that increases the blood concentration of CRP, regular physical activity reduces it in both men and women regardless of their age [35]. We found in our study that hs-CRP concentration measured post-run was significantly elevated, by as much as 382%. Interestingly, a similar increase in the level of hs-CRP occurred after 12 hours of a 48-h run in our earlier study [23]. Significantly higher concentrations of hs-CRP are also recorded in the male and female marathon runners [36], and for recreational runners after a marathon or a half-marathon [37]. Long-distance running increases blood supply to muscles, the cardiopulmonary system and the skin while curtailing the amount of blood supplied to the gut [12, 15]. Intestinal hypoperfusion associated with the redistribution of blood away from the splanchnic area leads to rapid reperfusion resulting in inflammation and metabolic stress that may disrupt the integrity of the intestinal barrier [12, 15, 38].

The main function of the intestinal barrier is to selectively absorb and secrete substances while preventing the entry of harmful microparticles and microorganisms to the bloodstream [39]. Its key element is a single layer of epithelial cells, mainly enterocytes [40, 41], which are vulnerable to ischemic events and hypoxia and can be damaged by a reduced oxygen inflow to the intestinal epithelium during exercise [12, 18]. The location of I-FABP in the mature epithelium of villi facilitates its leakage into the bloodstream from enterocytes after intestinal mucosa injury [42, 43]. The correlation between I-FABP and exercise-associated splanchnic hypoperphysion and subsequent ischemia is well documented [9, 12]. Sixty minutes of running [44] or cycling [45] at 70% VO_2max_ or 30 minutes of resistance exercise [46] have been found to cause a significant increase in the concentration of I-FABP. In our study, a significantly higher serum concentration of I-FABP was observed after the run (+ 134%). A similar increase (+ 156%) has been reported for 17 runners who had run for 90 min at 80% of their best 10 km race speed [43].

One of the biomarkers we assessed in our study was zonulin, a protein modulating tight junction activity, the concentration of which can be measured extracellularly in human serum [47]. According to research, intense and long-lasting effort [48], running for 90 min [49], and four weeks of treadmill exercise [50] can significantly raise the concentration of zonulin. The 17% increase in the concentration of zonulin recorded in our study after the run points to greater intestinal permeability in the participants. It must be noted, however, that the serum zonulin concentrations yielded by the commercial ELISA tests need to be interpreted with caution, because comparisons between patients with GI dysfunction and healthy persons have shown that the tests fail to detect prehaptoglobin-2 [21].

### Study limitation

This study has two main limitations. Firstly, we used a non-random sampling method to select participants. Secondly, neither the macronutrient intake nor the hydration of the participants was monitored during the run.

## Conclusion

Our hypothesis that a strenuous 12-h run would induce metabolic stress in middle-aged amateur runners was confirmed by disturbed prooxidant-antioxidant balance and elevated levels of inflammation and intestinal permeability biomarkers in the study participants. However, more research is necessary and a randomized sample of participants to ascertain this result.

## Supporting information

S1 Dataset (XLS)

## References

[1] Knechtle B, Nikolaidis PT. Physiology and Pathophysiology in Ultra-Marathon Running. Front Physiol [Internet]. 2018 [cited 2020 Nov 10];9. Available from: https://www.frontiersin.org/articles/10.3389/fphys.2018.00634/full. 10.3389/fphys.2018.00634 29910741 PMC5992463

[2] Powers SK, Radak Z, Ji LL. Exercise-induced oxidative stress: past, present and future. J Physiol. 2016;594(18):5081–92. 10.1113/JP270646 26893258 PMC5023699

[3] Ebele IJ, Jenniger IA, Nnabugo EC, Sidney OI, Chibuike OK, Chukwuma OO, et al. Oxidative stress/lipid peroxidation and antioxidant enzymes in apparently healthy individuals involved in physical exercise. Asian J Med Sci. 2016 10 31;7(6):16–9.

[4] Pilch W, Tota Ł, Piotrowska A, Śliwicka E, Czerwińska-Ledwig O, Zuziak R, et al. Effects of Nordic Walking on Oxidant and Antioxidant Status: Levels of Calcidiol and Proinflammatory Cytokines in Middle-Aged Women. Zhang Y, editor. Oxid Med Cell Longev. 2018 3 20;2018:6468234. 10.1155/2018/6468234 29743982 PMC5884206

[5] Kocabaş R, Namiduru E, Bagçeci A, Erenler A, Karakoç Ö, Örkmez M, et al. The acute effects of interval exercise on oxidative stress and antioxidant status in volleyball players. J Sports Med Phys Fitness. 2016 9 22;58. 10.23736/S0022-4707.16.06720-7 27653155

[6] Mittal M, Siddiqui MR, Tran K, Reddy SP, Malik AB. Reactive Oxygen Species in Inflammation and Tissue Injury. Antioxid Redox Signal. 2013 9 2;20(7):1126–67. 10.1089/ars.2012.5149 23991888 PMC3929010

[7] Sproston NR, Ashworth JJ. Role of C-Reactive Protein at Sites of Inflammation and Infection. Front Immunol [Internet]. 2018 [cited 2020 Apr 6];9. Available from: https://www.frontiersin.org/articles/10.3389/fimmu.2018.00754/full. 10.3389/fimmu.2018.00754 29706967 PMC5908901

[8] Pals KL, Chang R-T, Ryan AJ, Gisolfi CV. Effect of running intensity on intestinal permeability. J Appl Physiol. 1997 2 1;82(2):571–6. 10.1152/jappl.1997.82.2.571 9049739

[9] Costa RJS, Snipe RMJ, Kitic CM, Gibson PR. Systematic review: exercise-induced gastrointestinal syndrome—implications for health and intestinal disease. Aliment Pharmacol Ther. 2017;46(3):246–65. 10.1111/apt.14157 28589631

[10] Halvorsen FA, Lyng J, Glomsaker T, Ritland S. Gastrointestinal disturbances in marathon runners. Br J Sports Med. 1990 12 1;24(4):266–8. 10.1136/bjsm.24.4.266 2097027 PMC1478906

[11] Simons SM, Kennedy RG. Gastrointestinal problems in runners. Curr Sports Med Rep. 2004 3 1;3(2):112–6. 10.1249/00149619-200404000-00011 14980141

[12] van Wijck K, Lenaerts K, Grootjans J, Wijnands KAP, Poeze M, van Loon LJC, et al. Physiology and pathophysiology of splanchnic hypoperfusion and intestinal injury during exercise: strategies for evaluation and prevention. Am J Physiol-Gastrointest Liver Physiol. 2012 4 19;303(2):G155–68. 10.1152/ajpgi.00066.2012 22517770

[13] de Oliveira EP, Burini RC, Jeukendrup A. Gastrointestinal Complaints During Exercise: Prevalence, Etiology, and Nutritional Recommendations. Sports Med. 2014 5 1;44(1):79–85. 10.1007/s40279-014-0153-2 PMC4008808 24791919

[14] Derikx JPM, Matthijsen RA, de Bruïne AP, van Bijnen AA, Heineman E, van Dam RM, et al. Rapid Reversal of Human Intestinal Ischemia-Reperfusion Induced Damage by Shedding of Injured Enterocytes and Reepithelialisation. PLoS ONE [Internet]. 2008 10 17 [cited 2020 Apr 6];3(10). Available from: https://www.ncbi.nlm.nih.gov/pmc/articles/PMC2561292/. 10.1371/journal.pone.0003428 18927609 PMC2561292

[15] van Wijck K, Lenaerts K, van Loon LJC, Peters WHM, Buurman WA, Dejong CHC. Exercise-Induced Splanchnic Hypoperfusion Results in Gut Dysfunction in Healthy Men. PLoS ONE [Internet]. 2011 7 21 [cited 2020 Apr 6];6(7). Available from: https://www.ncbi.nlm.nih.gov/pmc/articles/PMC3141050/. 10.1371/journal.pone.0022366 21811592 PMC3141050

[16] Funaoka H, Kanda T, Fujii H. [Intestinal fatty acid-binding protein (I-FABP) as a new biomarker for intestinal diseases]. Rinsho Byori. 2010 2;58(2):162–8. 20229815

[17] Vanuytsel T, Vermeire S, Cleynen I. The role of Haptoglobin and its related protein, Zonulin, in inflammatory bowel disease. Tissue Barriers. 2013 12 1;1(5):e27321. 10.4161/tisb.27321 24868498 PMC3943850

[18] Blikslager AT. Life in the Gut Without Oxygen: Adaptive Mechanisms and Inflammatory Bowel Disease. Gastroenterology. 2008 1 1;134(1):346–8. 10.1053/j.gastro.2007.11.049 18166362

[19] Fasano A, Uzzau S. Modulation of intestinal tight junctions by Zonula occludens toxin permits enteral administration of insulin and other macromolecules in an animal model. J Clin Invest. 1997 3 15;99(6):1158–64. 10.1172/JCI119271 9077522 PMC507928

[20] Fasano A. Zonulin and Its Regulation of Intestinal Barrier Function: The Biological Door to Inflammation, Autoimmunity, and Cancer. Physiol Rev. 2011 1 1;91(1):151–75. 10.1152/physrev.00003.2008 21248165

[21] Ajamian M, Steer D, Rosella G, Gibson PR. Serum zonulin as a marker of intestinal mucosal barrier function: May not be what it seems. PLoS ONE [Internet]. 2019 1 14 [cited 2020 Apr 6];14(1). Available from: https://www.ncbi.nlm.nih.gov/pmc/articles/PMC6331146/. 10.1371/journal.pone.0210728 PMC6331146 30640940

[22] Mrakic-Sposta S, Gussoni M, Moretti S, Pratali L, Giardini G, Tacchini P, et al. Effects of Mountain Ultra-Marathon Running on ROS Production and Oxidative Damage by Micro-Invasive Analytic Techniques. PLOS ONE. 2015 11 5;10(11):e0141780. 10.1371/journal.pone.0141780 26540518 PMC4634988

[23] Kłapcińska B, Waśkiewicz Z, Chrapusta SJ, Sadowska-Krępa E, Czuba M, Langfort J. Metabolic responses to a 48-h ultra-marathon run in middle-aged male amateur runners. Eur J Appl Physiol. 2013 11 1;113(11):2781–93. 10.1007/s00421-013-2714-8 24002469 PMC3824198

[24] Sehovic E, Knechtle B, Rüst CA, Rosemann T. 12-hour ultra-marathons—Increasing worldwide participation and dominance of Europeans. J Hum Sport Exerc. 2013;8(4):932–53.

[25] Sánchez-Rodríguez MA, Mendoza-Núñez VM. Oxidative Stress Indexes for Diagnosis of Health or Disease in Humans. Oxid Med Cell Longev. 2019;2019:4128152. 10.1155/2019/4128152 31885788 PMC6899293

[26] Van Beaumont W. Evaluation of hemoconcentration from hematocrit measurements. J Appl Physiol. 1972 5 1;32(5):712–3. 10.1152/jappl.1972.32.5.712 5038863

[27] Kraemer RR, Brown BS. Alterations in plasma-volume-corrected blood components of marathon runners and concomitant relationship to performance. Eur J Appl Physiol. 1986 11 1;55(6):579–84. 10.1007/BF00423200 3096725

[28] Dunlap WP, Cortina JM, Vaslow JB, Burke MJ. Meta-analysis of experiments with matched groups or repeated measures designs. Psychol Methods. 1996;1(2):170–7.

[29] Faul F, Erdfelder E, Lang A-G, Buchner A. G*Power 3: A flexible statistical power analysis program for the social, behavioral, and biomedical sciences. Behav Res Methods. 2007 5;39(2):175–91. 10.3758/bf03193146 17695343

[30] Machefer G, Groussard C, Rannou-Bekono F, Zouhal H, Faure H, Vincent S, et al. Extreme Running Competition Decreases Blood Antioxidant Defense Capacity. J Am Coll Nutr. 2004 8 1;23(4):358–64. 10.1080/07315724.2004.10719379 15310740

[31] Hussain T, Tan B, Yin Y, Blachier F, Tossou MCB, Rahu N. Oxidative Stress and Inflammation: What Polyphenols Can Do for Us? Rupasinghe V, editor. Oxid Med Cell Longev. 2016 9 22;2016:7432797. 10.1155/2016/7432797 27738491 PMC5055983

[32] Black S, Kushner I, Samols D. C-reactive Protein. J Biol Chem. 2004 11 19;279(47):48487–90. 10.1074/jbc.R400025200 15337754

[33] Vermeire S, Van Assche G, Rutgeerts P. The role of C-reactive protein as an inflammatory marker in gastrointestinal diseases. Nat Clin Pract Gastroenterol Hepatol. 2005 12;2(12):580–6. 10.1038/ncpgasthep0359 16327837

[34] Clos TWD. Function of C-reactive protein. Ann Med. 2000 1 1;32(4):274–8. 10.3109/07853890009011772 10852144

[35] Fedewa MV, Hathaway ED, Ward-Ritacco CL. Effect of exercise training on C reactive protein: a systematic review and meta-analysis of randomised and non-randomised controlled trials. Br J Sports Med. 2017 4 1;51(8):670–6. 10.1136/bjsports-2016-095999 27445361

[36] Weight LM, Alexander D, Jacobs P. Strenuous exercise: analogous to the acute-phase response? Clin J Sport Med. 1992 4;2(2):150. 10.1042/cs0810677 1721863

[37] Niemelä M, Kangastupa P, Niemelä O, Bloigu R, Juvonen T. Acute Changes in Inflammatory Biomarker Levels in Recreational Runners Participating in a Marathon or Half-Marathon. Sports Med—Open. 2016 3 2;2(1):21. 10.1186/s40798-016-0045-0 27747777 PMC5005625

[38] Costa K, Soares A, Wanner S, Santos R dos, Fernandes S, Martins F, et al. L-Arginine Supplementation Prevents Increases in Intestinal Permeability and Bacterial Translocation in Male Swiss Mice Subjected to Physical Exercise under Environmental Heat Stress1-3. J Nutr. 2014 2;144(2):218–23. 10.3945/jn.113.183186 24259555

[39] Takiishi T, Fenero CIM, Câmara NOS. Intestinal barrier and gut microbiota: Shaping our immune responses throughout life. Tissue Barriers. 2017 10 2;5(4):e1373208. 10.1080/21688370.2017.1373208 28956703 PMC5788425

[40] Vancamelbeke M, Vermeire S. The intestinal barrier: a fundamental role in health and disease. Expert Rev Gastroenterol Hepatol. 2017 9 2;11(9):821–34. 10.1080/17474124.2017.1343143 28650209 PMC6104804

[41] Chelakkot C, Ghim J, Ryu SH. Mechanisms regulating intestinal barrier integrity and its pathological implications. Exp Mol Med. 2018 8 16;50(8):1–9. 10.1038/s12276-018-0126-x 30115904 PMC6095905

[42] Derikx JPM, Vreugdenhil ACE, Van den Neucker AM, Grootjans J, van Bijnen AA, Damoiseaux JGMC, et al. A Pilot Study on the Noninvasive Evaluation of Intestinal Damage in Celiac Disease Using I-FABP and L-FABP. J Clin Gastroenterol. 2009 9;43(8):727–33. 10.1097/MCG.0b013e31819194b0 19359998

[43] Adriaanse MPM, Tack GJ, Passos VL, Damoiseaux JGMC, Schreurs MWJ, Wijck K van, et al. Serum I-FABP as marker for enterocyte damage in coeliac disease and its relation to villous atrophy and circulating autoantibodies. Aliment Pharmacol Ther. 2013;37(4):482–90. 10.1111/apt.12194 23289539

[44] Sessions J, Bourbeau K, Rosinski M, Szczygiel T, Nelson R, Sharma N, et al. Carbohydrate gel ingestion during running in the heat on markers of gastrointestinal distress. Eur J Sport Sci. 2016 11 16;16(8):1064–72. 10.1080/17461391.2016.1140231 26841003

[45] Van Wijck K, Lenaerts K, Van Bijnen AA, Boonen B, Van Loon LJC, Dejong CHC, et al. Aggravation of Exercise-Induced Intestinal Injury by Ibuprofen in Athletes. Med Sci Sports Exerc. 2012 12;44(12):2257–62. 10.1249/MSS.0b013e318265dd3d 22776871

[46] van Wijck K, Pennings B, van Bijnen AA, Senden JMG, Buurman WA, Dejong CHC, et al. Dietary protein digestion and absorption are impaired during acute postexercise recovery in young men. Am J Physiol-Regul Integr Comp Physiol. 2013 1 2;304(5):R356–61. 10.1152/ajpregu.00294.2012 23283940

[47] Tripathi A, Lammers KM, Goldblum S, Shea-Donohue T, Netzel-Arnett S, Buzza MS, et al. Identification of human zonulin, a physiological modulator of tight junctions, as prehaptoglobin-2. Proc Natl Acad Sci. 2009 9 29;106(39):16799–804. 10.1073/pnas.0906773106 19805376 PMC2744629

[48] Tota Ł, Piotrowska A, Pałka T, Morawska M, Mikuľáková W, Mucha D, et al. Muscle and intestinal damage in triathletes. PLOS ONE. 2019 1 18;14(1):e0210651. 10.1371/journal.pone.0210651 30657773 PMC6338373

[49] Karhu E, Forsgård RA, Alanko L, Alfthan H, Pussinen P, Hämäläinen E, et al. Exercise and gastrointestinal symptoms: running-induced changes in intestinal permeability and markers of gastrointestinal function in asymptomatic and symptomatic runners. Eur J Appl Physiol. 2017 12 1;117(12):2519–26. 10.1007/s00421-017-3739-1 29032392 PMC5694518

[50] Shin HE, Kwak SE, Zhang DD, Lee J, Yoon KJ, Cho HS, et al. Effects of treadmill exercise on the regulation of tight junction proteins in aged mice. Exp Gerontol. 2020 11 1;141:111077. 10.1016/j.exger.2020.111077 32898618

